# Supplementary material for: SpliceSelectNet: a hierarchical Transformer-based deep learning model for splice site prediction
Source: Nucleic Acids Res. 2026 Jun 22;54(12):gkag625. doi: 10.1093/nar/gkag625 (PMC13284721; doi:10.1093/nar/gkag625)
Supplement: gkag625_Supplemental_File [file gkag625_supplemental_file.pdf]

Supplementary Material for "SpliceSelectNet: A Hierarchical Transformer-Based Deep Learning Model for Splice Site Prediction"

Yuna Miyachi and Kenta Nakai

Supplementary Figures

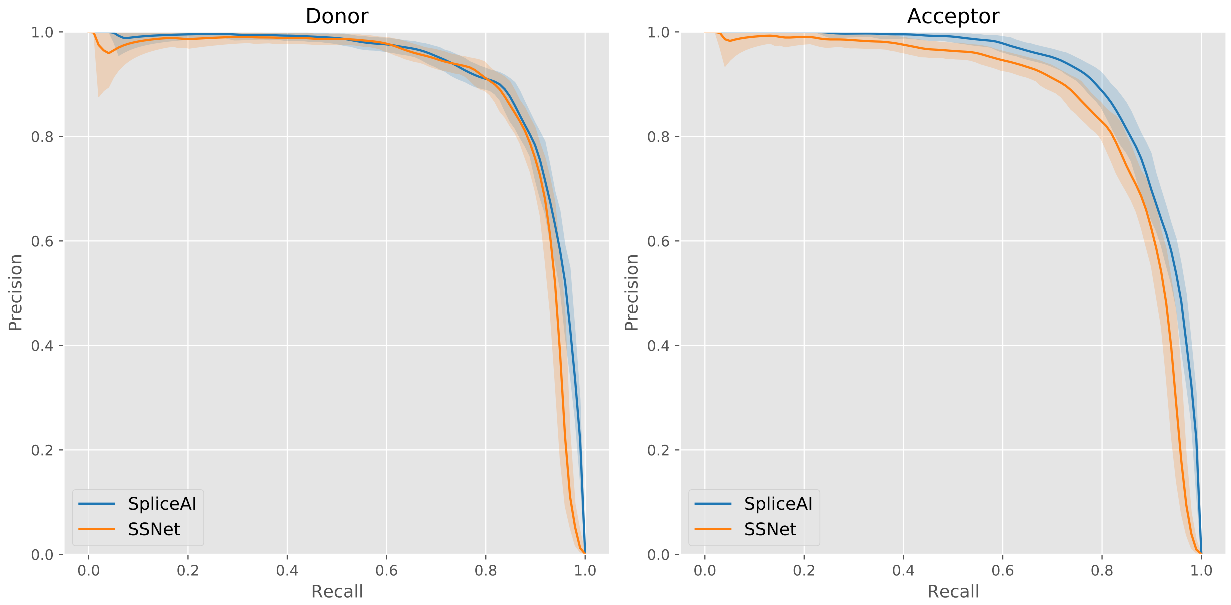

Supplementary Figure S1: Performance of SSNet_base and SpliceAI on lincRNA. PR curves are shown for donor and acceptor sites. Shaded regions indicate 95% confidence intervals estimated by stratified bootstrap resampling.

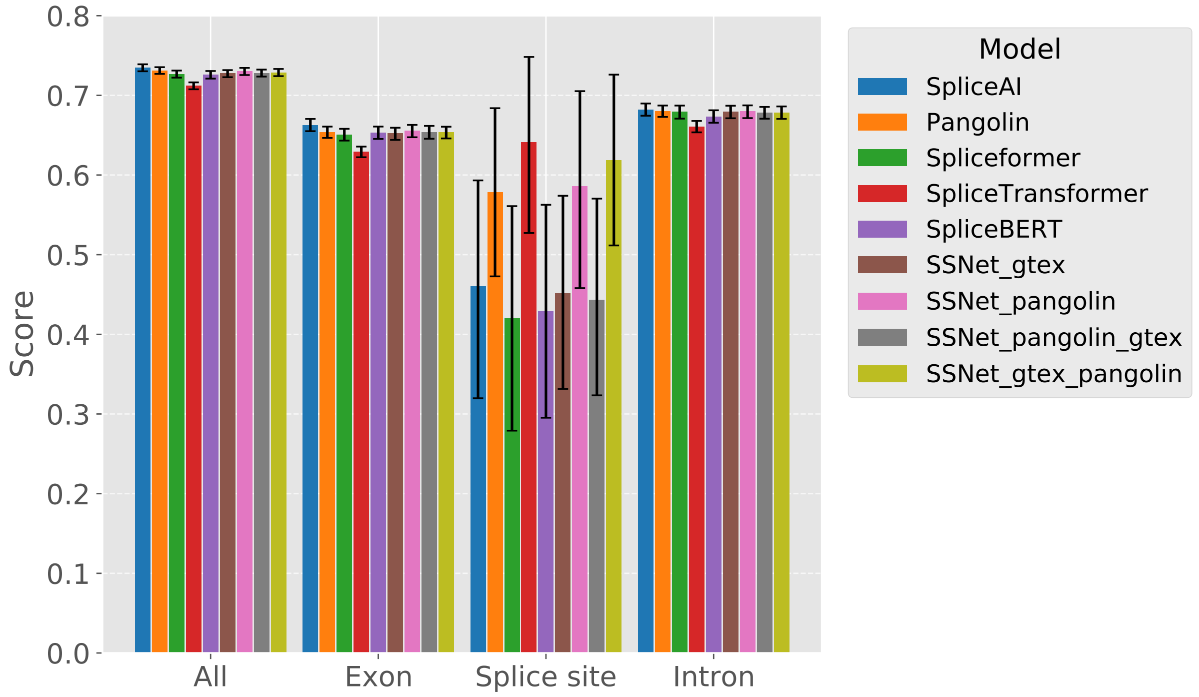

Supplementary Figure S2: Performance of all SSNet variants and state-of-the-art models on SpliceVarDB. AUROC values are shown for all variants combined and stratified by variant location (Exon, SpliceSite, Intron). SSNet variants include SSNet_gtex, SSNet_pangolin, SSNet_gtex_pangolin, and SSNet_pangolin_gtex. Other models include SpliceAI, Pangolin, Spliceformer, SpliceTransformer, and SpliceBERT. Error bars indicate 95% confidence intervals estimated by stratified bootstrap resampling.

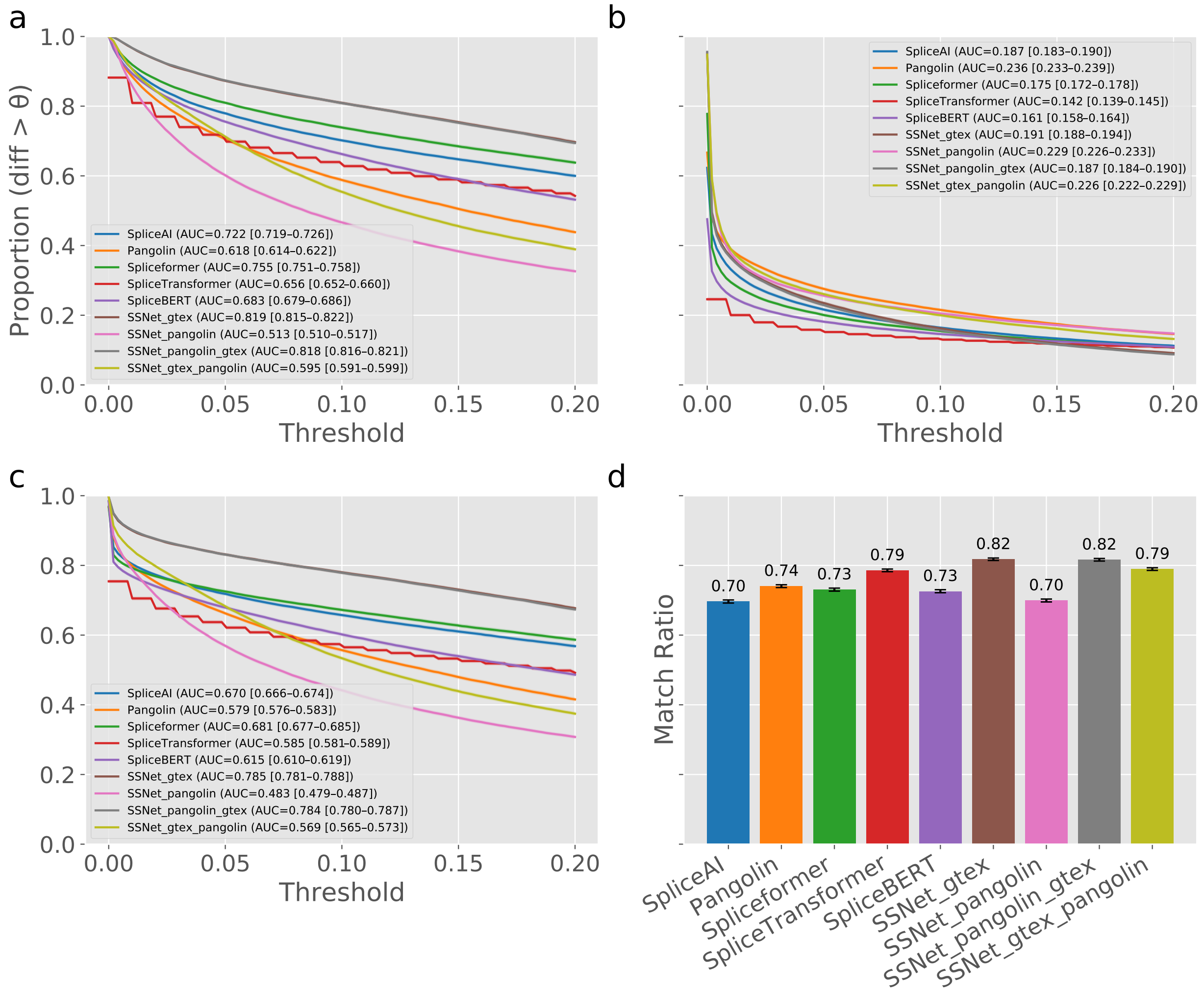

Supplementary Figure S3: Performance of all SSNet variants and state-of-the-art models on SSCVDB. Proportion of predicted splicing changes exceeding thresholds (0.0–0.2) is shown for (a) the entire sequence, (b) original splice sites (Hijacked_SS), and (c) newly created splice sites (Primary_SS) in SSCVDB. (d) The probability that the maximum predicted change occurs at Primary_SS. Error bars indicate 95% confidence intervals estimated by stratified bootstrap resampling.

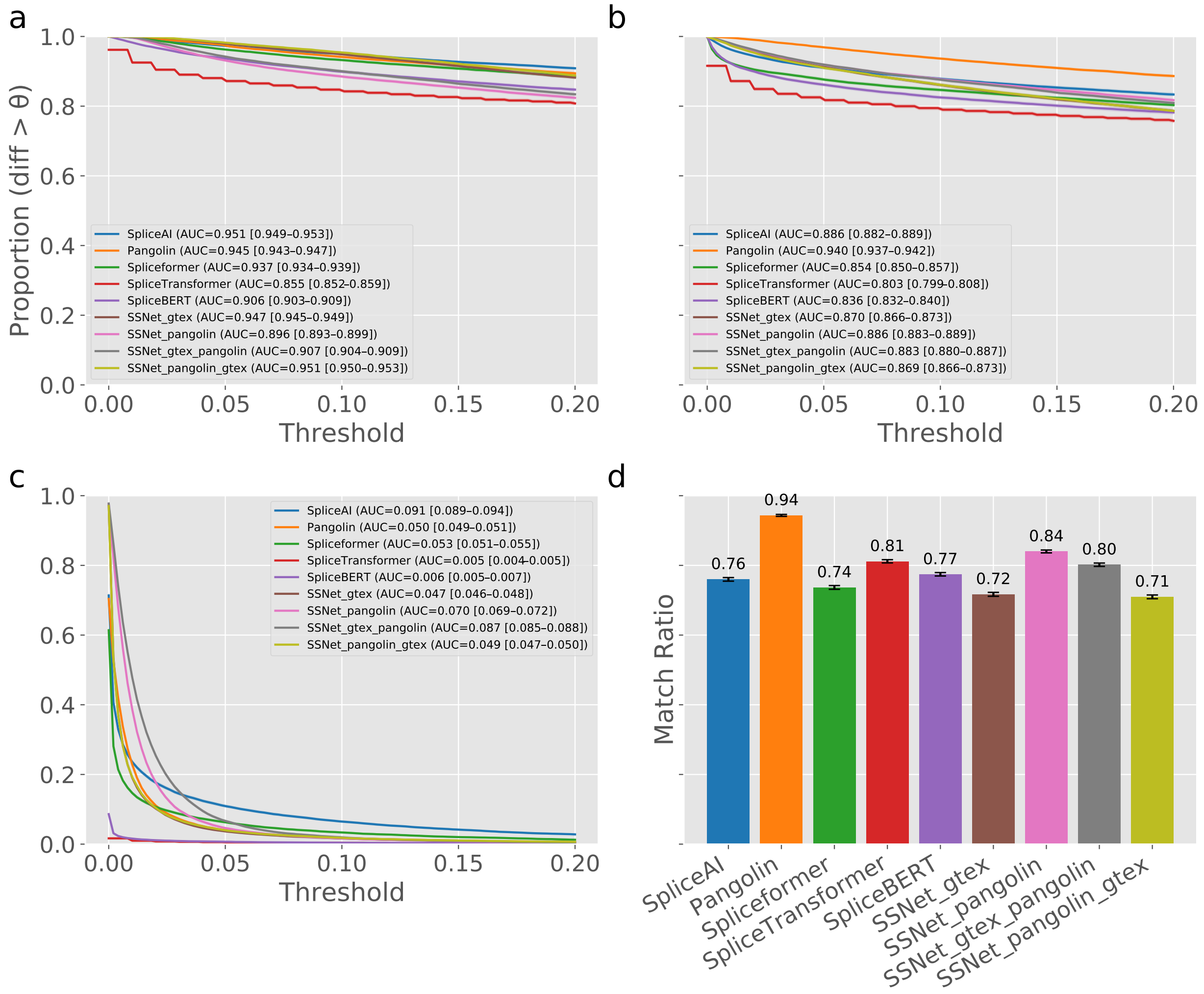

Supplementary Figure S4: Intron retention detection performance of all SSNet variants and state-of-the-art models on IRAVDB. Proportion of predicted splicing changes exceeding thresholds (0.0–0.2) is shown for (a) the entire sequence, (b) target splice sites (target_pos), and (c) their partner splice sites (partner_pos). (d) The probability that the maximum predicted change occurs at target_pos. Error bars indicate 95% confidence intervals estimated by stratified bootstrap resampling.

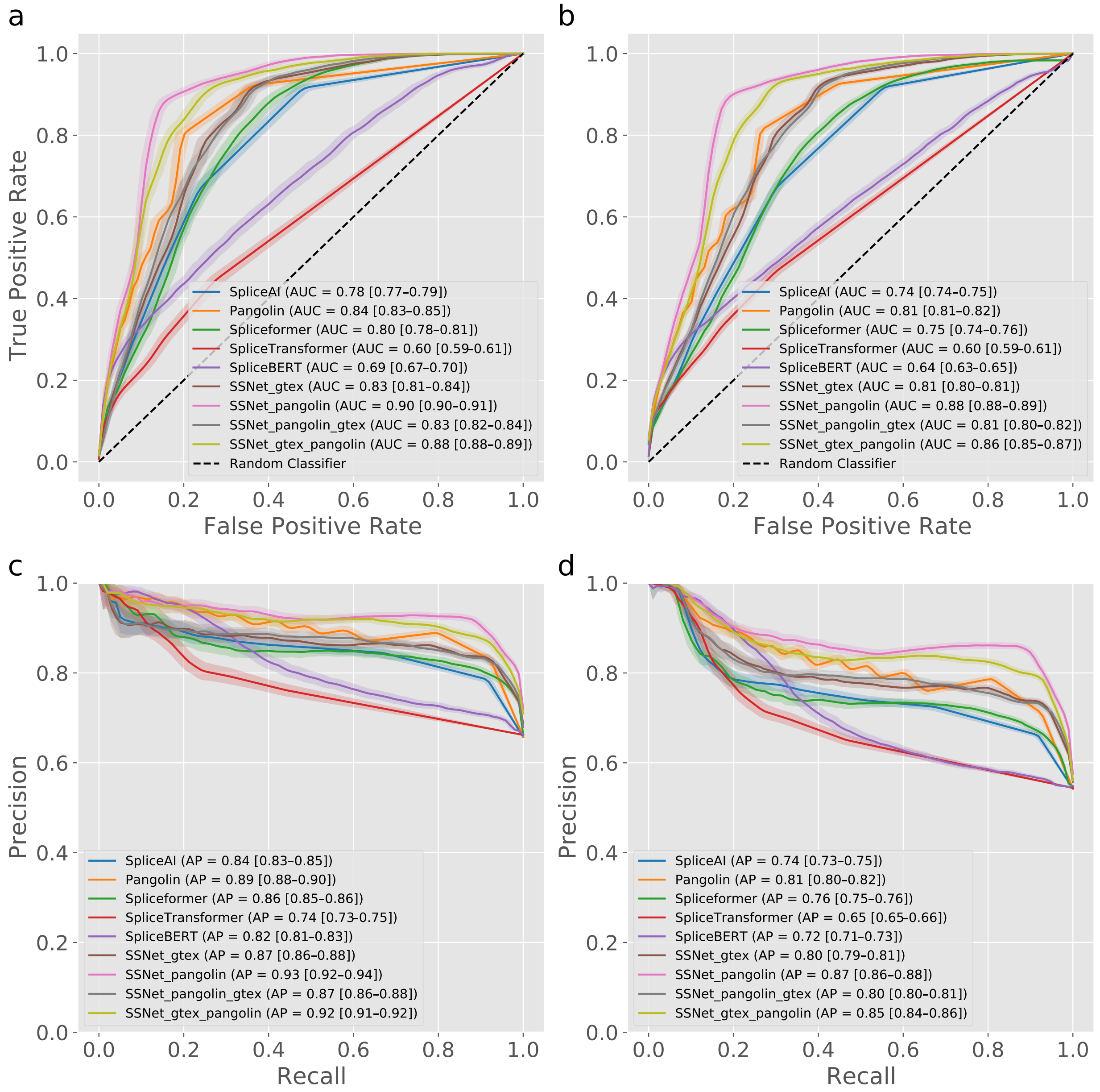

Supplementary Figure S5: Performance of all SSNet variants and state-of-the-art models on the BRCA dataset. Shaded regions indicate 95% confidence intervals estimated by stratified bootstrap resampling. (a) ROC curves for expert label. (b) ROC curves for all label. (c) PR curves for expert label. (d) PR curves for all label.

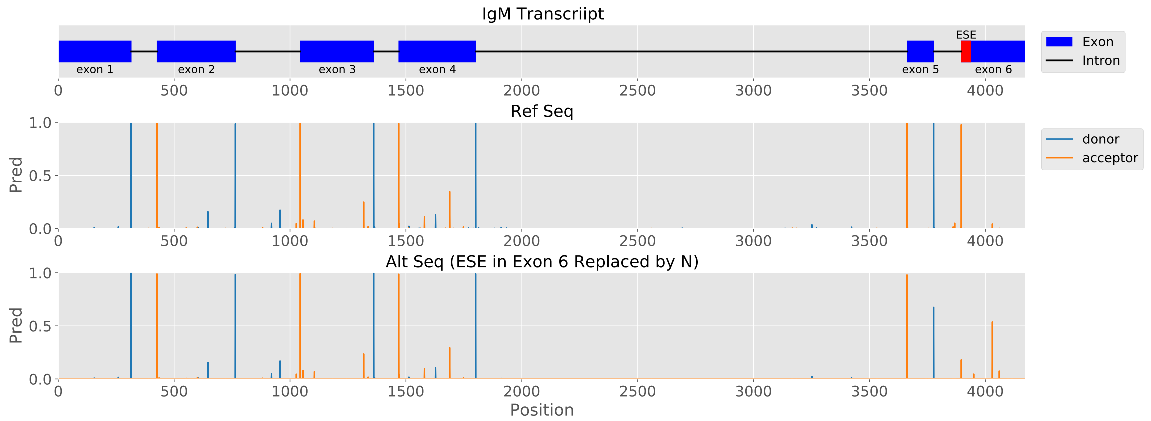

Supplementary Figure S6: SpliceAI predictions for the mouse IgM gene. Top: gene structure with introns (black lines) and exons (blue boxes); the 42 bp ESE at the 5' end of Exon 6 is highlighted in red. Bottom: predicted donor (blue) and acceptor (orange) probabilities for the wild-type sequence and with the ESE region masked with "N". Masking the ESE abolishes the acceptor site at the 5' end of Exon 6 (red box).

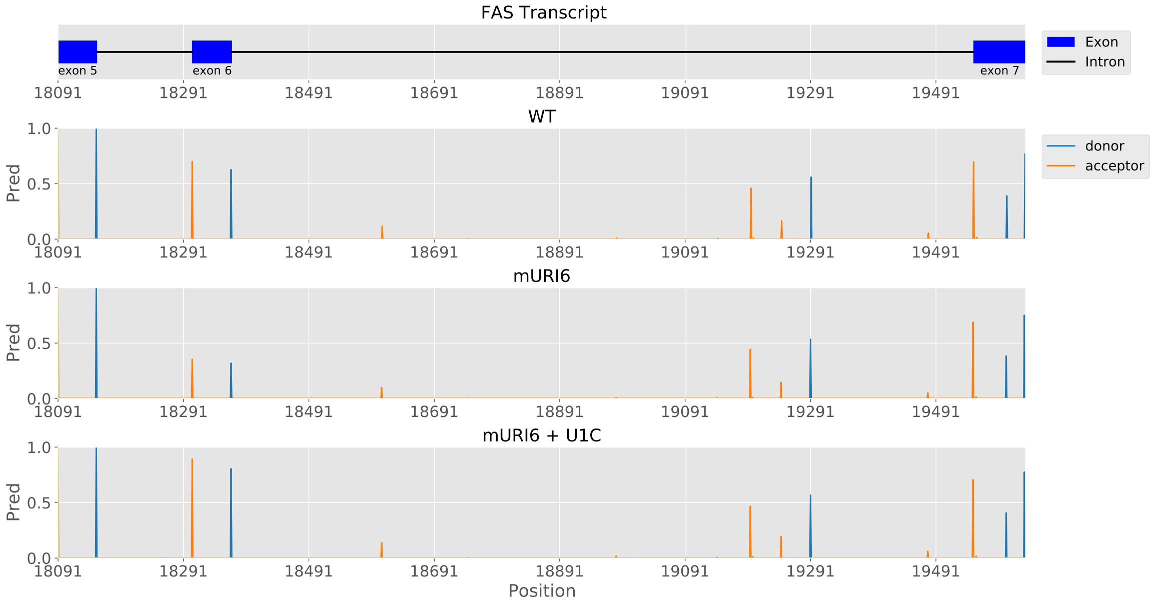

Supplementary Figure S7: SpliceAI predictions for FAS wild-type, mURI6, and mURI6+U1C sequences. The red boxes indicate regions where splice site probabilities are altered by mutations.

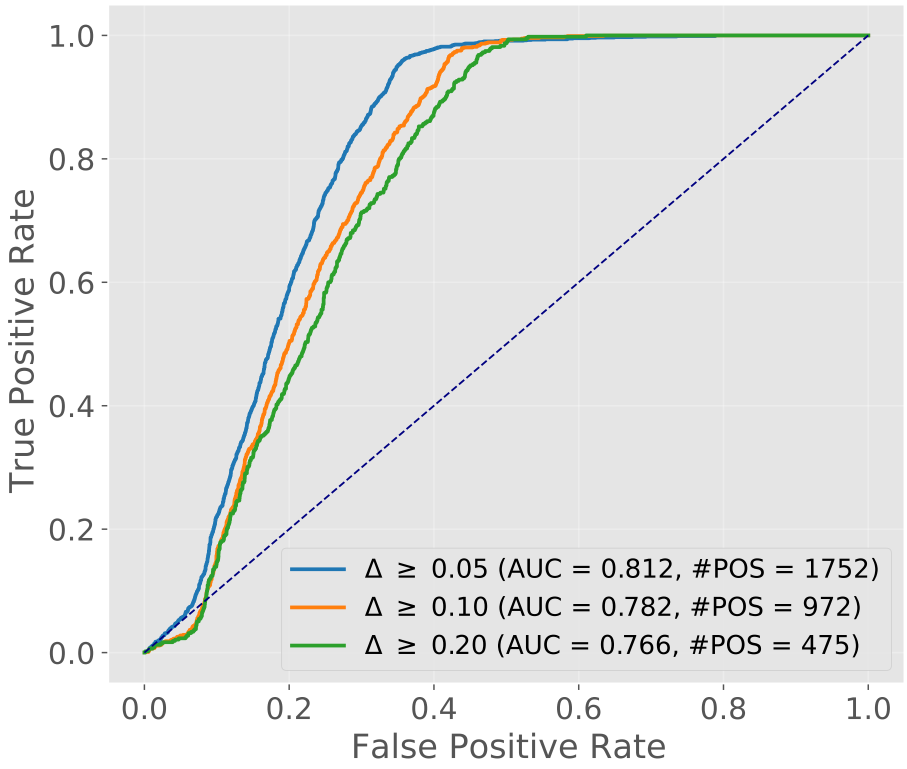

Supplementary Figure S8: ROC curves for prediction difference values based on attention z-score at multiple thresholds (0.05, 0.1, 0.2).

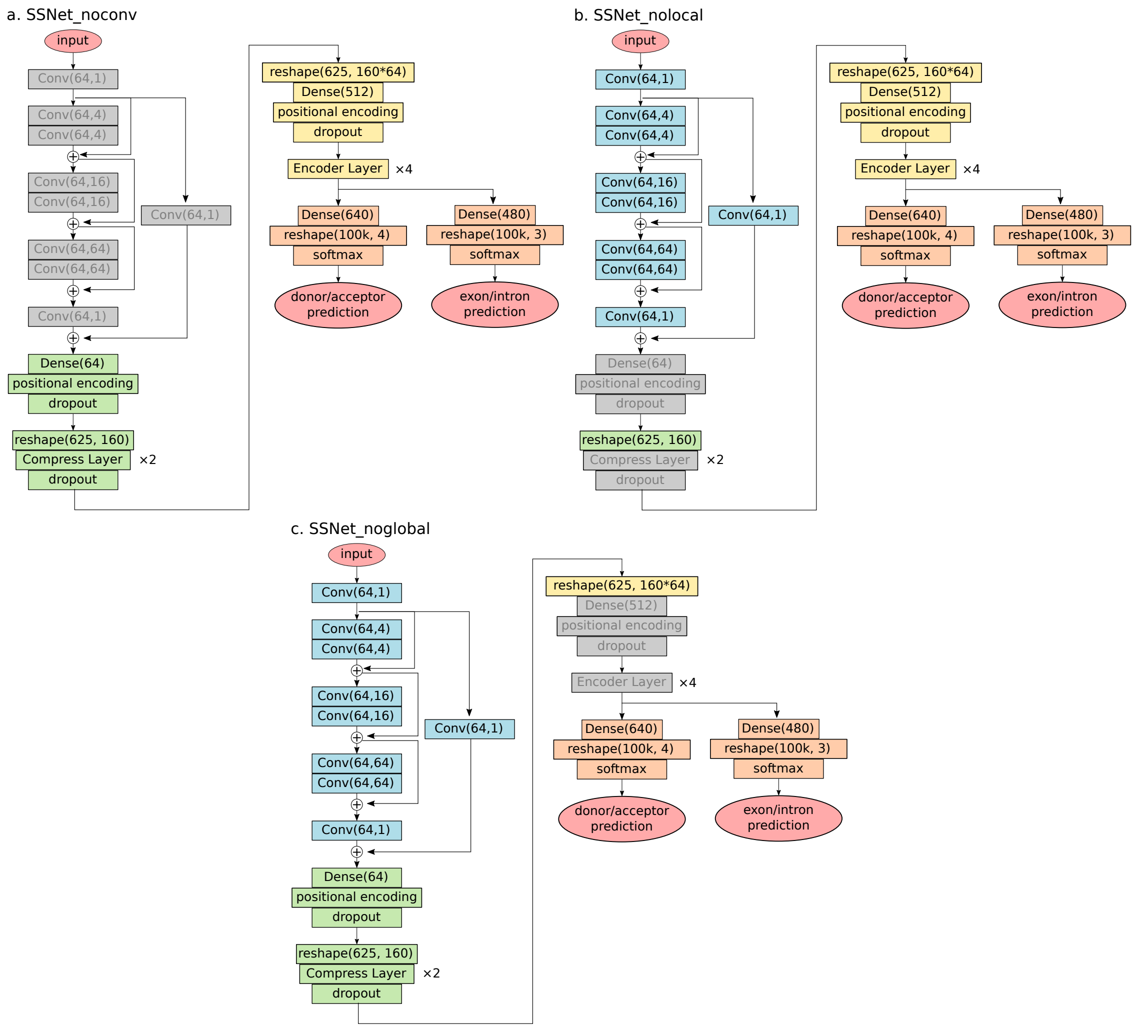

Supplementary Figure S9: Layer-wise ablation study of SSNet architecture. This figure highlights which components of the model (Convolutional layers, Local Attention, Global Attention) were used or commented out in the ablation experiments (SSNet_noconv, SSNet_nolocal, SSNet_noglobal). Performance metrics are reported in Table 1 of the main text.

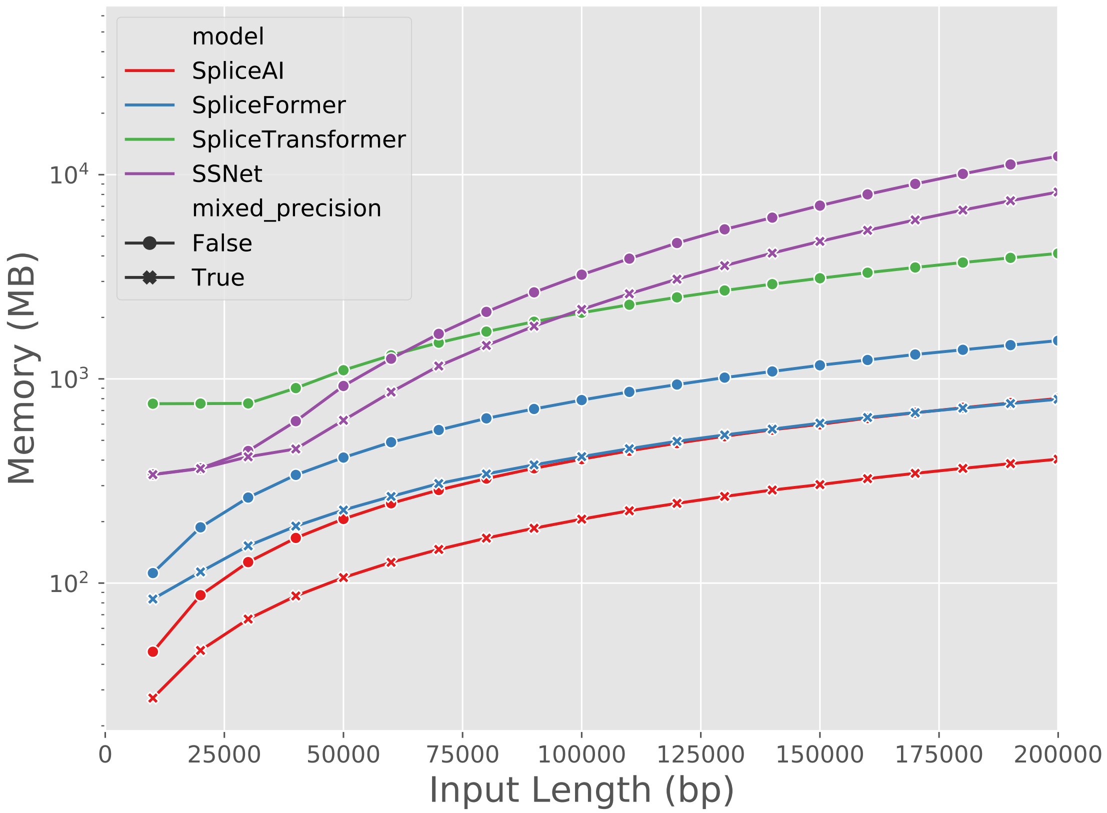

Supplementary Figure S10: Memory consumptions of different models (SpliceAI, Spliceformer, SpliceTransformer, SSNet) across various input sequence length.

Supplementary Tables

Supplementary Table S1: Training Data Summary. Summary of datasets used for model training. Donor and acceptor counts are derived from labeled splice sites, and for Pangolin, the Average SSE (donor/acceptor) is reported.

Dataset	#Genes	#Donor	#Acceptor	Ave SSE (donor)	Ave SSE (acceptor)
Gencode	13384	130795	130795	-	-
GTEx	13385	193699	197804	-	-
Pangolin	13384	158621	156361	0.612	0.606

Supplementary Table S2: Validation Data Summary for SVDB. The number of genes and positive/negative variants for all regions and region-specific (Exon, SpliceSite, Intron) are shown. Label 3 shows splice-altering variants, Label 2 shows low frequency splice-altering ones, and Label 1 shows not splice-altering ones.

Region	#Variants	#Genes	#label=3	#label=2	#label=1
All	48104	7538	12943	24282	10804
Exon	22859	4544	3357	13697	5740
Splice Site	7334	4350	5800	1511	22
Intron	17911	3990	3786	9074	5042

Supplementary Table S3: Validation Data Summary for BRCA. The number of genes and positive/negative variants for expert and all labels are shown.

Label Type	# Variants	#Genes	#Pathogenic	#Benign	#Unknown
label_expert	72467	2	4909	2544	65014
label_all	72467	2	7966	6619	57882

Supplementary Table S4: Validation Results for SVDB. TP, FP, TN, FN counts at fixed threshold are provided, along with AUROC and AUPRC. Results are grouped by region.

Region	Model	#TP	#FP	#TN	#FN	AUROC	AUPRC
All	SpliceAI	14765	259	10620	22460	0.735	0.916
	Pangolin	14629	210	10668	22592	0.731	0.918
	Spliceformer	14596	303	10576	22629	0.727	0.913
	SpliceTransformer	13156	178	10701	24069	0.712	0.912
	SpliceBERT	13942	242	10637	23283	0.726	0.912
	SSNet_gtex	14145	256	10623	23080	0.728	0.913
	SSNet_pangolin	12455	94	10785	24770	0.730	0.915
	SSNet_gtex_pangolin	13013	103	10776	24212	0.728	0.915
	SSNet_pangolin_gtex	14252	258	10621	22973	0.728	0.913
Exon	SpliceAI	3557	115	5690	13497	0.663	0.865
	Pangolin	3519	105	5700	13535	0.654	0.866
	Spliceformer	3490	137	5668	13564	0.651	0.860
	SpliceTransformer	2763	77	5728	14291	0.629	0.855
	SpliceBERT	3256	126	5679	13798	0.653	0.860
	SSNet_gtex	3244	131	5674	13810	0.652	0.859
	SSNet_pangolin	2292	37	5768	14762	0.655	0.863
	SSNet_gtex_pangolin	2689	40	5765	14365	0.653	0.862
	SSNet_pangolin_gtex	3304	134	5671	13750	0.653	0.860
Splice Site	SpliceAI	7272	21	2	39	0.459	0.996
	Pangolin	7265	21	2	46	0.576	0.998
	Spliceformer	7274	23	0	37	0.424	0.995
	SpliceTransformer	7223	22	1	88	0.640	0.998
	SpliceBERT	7251	23	0	60	0.429	0.996
	SSNet_gtex	7281	21	2	30	0.458	0.997
	SSNet_pangolin	7250	21	2	61	0.585	0.998
	SSNet_gtex_pangolin	7254	21	2	57	0.618	0.998
	SSNet_pangolin_gtex	7280	21	2	31	0.442	0.996
Intron	SpliceAI	3936	123	4928	8924	0.682	0.865
	Pangolin	3845	84	4966	9011	0.680	0.870
	Spliceformer	3832	143	4908	9028	0.679	0.863
	SpliceTransformer	3170	79	4972	9690	0.661	0.861
	SpliceBERT	3435	93	4958	9425	0.673	0.860
	SSNet_gtex	3620	104	4947	9240	0.679	0.863
	SSNet_pangolin	2913	36	5015	9947	0.680	0.866
	SSNet_gtex_pangolin	3070	42	5009	9790	0.678	0.865
	SSNet_pangolin_gtex	3668	103	4948	9192	0.678	0.862

Supplementary Table S5: Validation Results for BRCA. TP, FP, TN, FN counts at fixed threshold are provided, along with AUROC and AUPRC.

Label	Model	#TP	#FP	#TN	#FN	AUROC	AUPRC
label_expert	SpliceAI	317	29	2470	4481	0.781	0.851
	Pangolin	517	19	2485	4311	0.844	0.897
	Spliceformer	328	18	2486	4581	0.796	0.855
	SpliceTransformer	337	19	2485	4567	0.599	0.773
	SpliceBERT	582	17	2487	4327	0.686	0.820
	SSNet_gtex	231	22	2482	4678	0.826	0.871
	SSNet_pangolin	239	5	2499	4670	0.904	0.929
	SSNet_gtex_pangolin	247	5	2499	4662	0.884	0.916
	SSNet_pangolin_gtex	256	26	2478	4653	0.831	0.874
label_all	SpliceAI	869	133	6397	6853	0.744	0.756
	Pangolin	1170	120	6440	6607	0.813	0.820
	Spliceformer	786	114	6452	7173	0.750	0.755
	SpliceTransformer	833	77	6485	6994	0.601	0.686
	SpliceBERT	1150	74	6492	6809	0.637	0.719
	SSNet_gtex	797	67	6499	7162	0.805	0.800
	SSNet_pangolin	769	23	6543	7190	0.883	0.872
	SSNet_gtex_pangolin	791	28	6538	7168	0.859	0.850
	SSNet_pangolin_gtex	824	86	6480	7135	0.809	0.802
